# Modelling and Simulation of the Drug Release from a Solid Dosage Form in the Human Ascending Colon: The Influence of Different Motility Patterns and Fluid Viscosities

**DOI:** 10.3390/pharmaceutics13060859

**Published:** 2021-06-10

**Authors:** Michael Schütt, Konstantinos Stamatopoulos, Hannah K. Batchelor, Mark J. H. Simmons, Alessio Alexiadis

**Affiliations:** 1 School of Chemical Engineering, University of Birmingham, Birmingham B15 2TT, UK; M.J.Simmons@bham.ac.uk; 2 Biopharmaceutics, Pharmaceutical Development, PDS, MST, RD Platform Technology & Science, GSK, David Jack Centre, Park Road, Ware SG12 0DP, UK; 3 Strathclyde Institute of Pharmacy and Biomedical Sciences, University of Strathclyde, 161 Cathedral Street, Glasgow G4 0RE, UK; hannah.batchelor@strath.ac.uk

**Keywords:** mathematical modelling, smoothed particle hydrodynamics (SPH), fluid dynamics, large intestine, colon, fluid–structure interactions, colonic drug delivery, tablet disintegration, drug release profile, spatiotemporal concentration profile

## Abstract

For colonic drug delivery, the ascending part of the colon is the most favourable site as it offers the most suitable environmental conditions for drug dissolution. Commonly, the performance of a drug formulation is assessed using standardised dissolution apparatus, which does not replicate the hydrodynamics and shear stress evoked by wall motion in the colon. In this work, computer simulations are used to analyse and understand the influence of different biorelevant motility patterns on the disintegration/drug release of a solid dosage form (tablet) under different fluid conditions (viscosities) to mimic the ascending colonic environment. Furthermore, the ability of the motility pattern to distribute the drug in the ascending colon luminal environment is analysed to provide data for a spatiotemporal concentration profile. The motility patterns used are derived from in vivo data representing different motility patterns in the human ascending colon. The applied motility patterns show considerable differences in the drug release rate from the tablet, as well as in the ability to distribute the drug along the colon. The drug dissolution/disintegration process from a solid dosage form is primarily influenced by the hydrodynamic and shear stress it experiences, i.e., a combination of motility pattern and fluid viscosity. Reduced fluid motion leads to a more pronounced influence of diffusion in the tablet dissolution process. The motility pattern that provoked frequent single shear stress peaks seemed to be more effective in achieving a higher drug release rate. The ability to simulate drug release profiles under biorelevant colonic environmental conditions provides valuable feedback to better understand the drug formulation and how this can be optimised to ensure that the drug is present in the desired concentration within the ascending colon.

## 1. Introduction

The number of people worldwide affected by colonic diseases such as inflammatory bowel disease (IBD) (i.e., Crohn’s disease (CD) and ulcerative colitis (UC)) has steadily increased from 3.7 million in 1990 to 6.8 million in 2017 [1]. Colon-specific drug delivery has been the focus of numerous studies in recent years (e.g., [2,3]), as it offers opportunities to improve the treatment of local diseases such as CD and UC while minimizing side effects at the same time [4].

The oral route, due to its convenience, is the primary method of administration for most medicines, including those that target the colon. Generally, the proximal colon is the targeted side for colonic drug delivery due to its more suitable environmental conditions (i.e., water availability for drug dissolution, fluid viscosities, less variable transit times), compared to the distal part of the colon [5,6,7,8]. Modified release (MR) dosage formulations are usually coated with a protective layer that dissolves on its way (e.g., pH dependent) to the colon so that the actual drug release takes place in the colonic environment [9]. To access the performance of a drug formulation, disintegration/dissolution tests are commonly performed using United States Pharmacopeia (USP) apparatus to mimic in vitro the complex in vivo process [10]. However, this simplified model does not replicate the hydrodynamics and the in vivo shear stresses, evoked by wall motion, which act on the MR formulation and influence the disintegration/dissolution process. Stamatopoulos et al. [11] developed an anatomically accurate in vitro model, the Dynamic Colon Model (DCM), where the hydrodynamics can be controlled using a hydraulic system and fluids with different compositions can be used to better replicate the human adult colon. To enable a more realistic environment compared with the USP and to support the data from in vitro tests performed with the DCM or even replace experimental work, [12] developed an in silico model which gives detailed insight into the hydrodynamics and mixing profiles occurring in the colonic environment at different conditions. Currently, the release from an MR formulation in vivo can only be visualised using Magnetic Resonance Imaging (MRI) or scintigraphy which is costly, time-consuming and not practical for product development and optimisation [13]. Moreover, the in silico models, including Physiologically Based Pharmacokinetic (PBPK) platforms such as GastroPlus^TM^ and Simcyp^®^, use simple first order forward transit rate model. Thus, the colonic environment is considered as a single well-mixed and homogenized compartment. Although, different transit times are used for different entities (e.g., tablet, pellets, and fine particles), however, they are not related to the motility, volumes and viscosity. Thus, a tablet will have a fixed transit time regardless of the motility, volumes and viscosity changes in vivo. Any variation (i.e., coefficient of variance, % CV) applied on the transit times is done just to reflect observed inter-subject variability.

However, this modelling strategy does not allow for intra-occasional and intra-subject variability. This is because motility patterns are not constant in each subject and there are limited in vivo studies that monitor motility and transit times of different entities at the same time. Moreover, the released and/or dissolved drug particles in GastroPlus^TM^ and Simcyp^®^ will be instantly and uniformly distributed throughout the colonic compartment which is in contradiction to findings from in vivo bioimaging studies. Further compartmentalization of the colon (i.e., splitting a single in silico compartment to many sub-compartments), will not provide a solution. In addition, shear stresses applied on the dosage form and on released drug particles are not used by GastroPlus^TM^ and Simcyp^®^. Instead, average velocities derived from Computational Fluid Dynamics (CFD) analysis of USP II are used. Thus, a non-biorelevant apparatus is used to describe in vivo hydrodynamics. Furthermore, these average velocities are not linked to transit times or to motility patterns. Thus, changes in transit times in these PBPK platforms does not mean changes in the fluid velocities.

Finally, although PBPK models may contain complex algorithms to account for the interconnection of the different organs/compartments, are simply first order models and they cannot reproduce multiphysical phenomena such as the complex interplay between, e.g., wall motion, fluid motion, fluid viscosity, particle-particle and particle-fluid interactions. This is the reason why PBPK platforms cannot utilize either in vivo studies providing motility patterns (i.e., wall motion) or pressure amplitudes and link all the components affecting hydrodynamics, e.g., fluid volumes, fluid viscosity, transit times, direction of fluid, and spatiotemporal distribution of, e.g., fluid velocities and shear forces according to the intestinal wall motion. Thus, advanced modelling techniques are required to provide an increase understanding of the behaviour of a dosage form in a complex and dynamically changing in vivo environment. Therefore, in this work an in silico model that replicates, both the in vivo colonic environment and the disintegration/dissolution process of a pharmaceutical formulation to provide the distribution of the released drug along the colon would be beneficial for pharmaceutical development.

In this study, five motility patterns were chosen to replicate in vivo motility: three different motility patterns identified in Stamatopoulos et al. [13] and Marciani et al. [14] plus two additional motility patterns with data from [15,16]. The influence of these five motility patterns on the disintegration/dissolution process of a solid dosage form (tablet) at different fluid viscosities are analysed and compared. The motility patterns in Stamatopoulos et al. [13] were derived from cine-MRI data of the cecum-ascending colon in healthy adult humans [14]. Additionally, we analyse the performance of the motility patterns in terms of the distribution of the dissolved drug along the colon at different fluid viscosities, providing data for a spatiotemporal concentration profile.

Effective drug therapy relies on the active pharmaceutical agent (API) being released from the solid oral dosage form and subsequently dissolving into the luminal fluid such that it can act locally on the receptor or traverse the membrane for systemic uptake. This rate of dissolution is a function of the formulation, the API and the conditions for dissolution. Simulation of the tablet dissolution within the colonic model provides further insights as a model that provides understanding of the in vivo performance of a drug formulation enables feedback early in development such that the drug product and manufacturing process can be optimised.

These new data also provide further information that can be used for the development of future drug formulations, as the different motility patterns in the colonic environment may have a crucial role in the disintegration/dissolution process of a solid dosage form. For the simulations, we use an approach similar to Schütt et al. [12], further optimised to replicate the haustra and with closed ends, so that the model is closer to real environmental conditions. We also implement a solid dosage form that can disintegrate/dissolve in the colonic fluid. The drug release/disintegration process of the tablet can be adapted to real tablet data (drug release/disintegration behaviour) and thus a large variety of different solid dosage forms can be replicated.

## 2. Methodology

### 2.1. Modelling Approach

The simulation technique used in this study is based on Discrete Multiphysics (DMP), a modelling technique already successfully used in Alexiadis et al. [17,18,19], Ariane et al. [20,21,22], Mohammed et al. [23] and Schütt et al. [12] to model human organs. DMP couples different particle-based modelling techniques such as Smoothed Particle Hydrodynamics (SPH), Lattice Spring Model (LSM), and the Discrete Element Method (DEM). The model in this study only accounts for SPH and LSM and it is related to Schütt et al. [12]. Theoretical background for DMP can be found in Alexiadis et al. [17,19], while for SPH and LSM it may be found in Ref. [24] and Refs. [25,26,27] respectively. The dissolution of the tablet is modelled according to the methodology in Rahmat et al. [28].

### 2.2. Colon-Model Geometry

**Figure 1:**
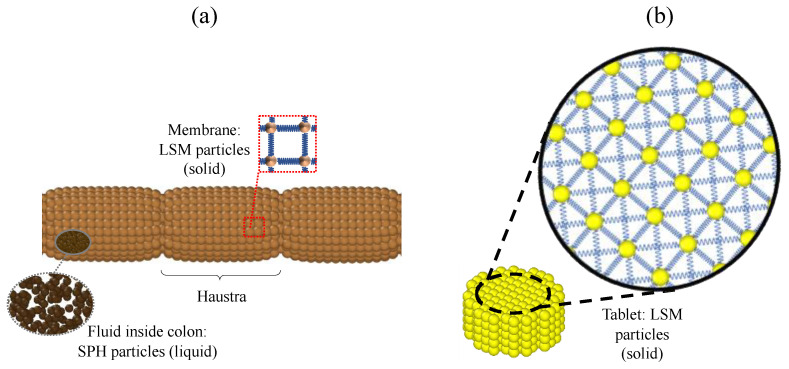
(**a**) Section of the flexible membrane, showing the colon’s haustra and the intestinal fluid inside the colon. The membrane is built of particles which are connected by a network of springs to achieve a flexible behaviour. (**b**) 3D sketch of the tablet. The particles representing the tablet are connected by linear and diagonal springs to obtain a solid behaviour.

Our 3D model represents an enlarged model of the human ascending colon (length scale 3:1, diameter 1:1). We developed five models that differ only in their operating conditions relating to the motility pattern and the number of sections (*haustra*) used. In all cases, the geometry of the model is the same: a cylindrical body with a total length of 6.0 × 10^−1^ m and an inner diameter of 4.0 × 10^−2^ m. All models are built with closed ends, which means that the fluid cannot exit the tube and back pressure is created when the fluid flow reaches the end. This mimics the presence of the *hepatic flexure* which is a sharp bend between the ascending and the transverse colon. In vivo studies observed that in the proximal colon, the majority of the waves only propagate over a short distance and commonly stop before the *hepatic flexure*, resulting in backflow/back pressure [29,30]. The model is divided into sections of equal size representing the colon’s *haustra* (Figure 1a). To simulate the conditions reported in [13] the number of sections differs from model to model.

There are a wide range of tablets of different sizes and shapes on the market, for the sake of simplicity, in this study, the geometry of the tablet is set as a cylinder with a diameter of 1.0 × 10^−2^ m and a height of 5.0 × 10^−3^ m (Figure 1b). For more details on the modelling of the tablet, see Section 2.3.4.

In the model, SPH is used for the fluid and LSM for the elastic membrane and the solid tablet. During the tablet dissolution process, fluid diffuses into the tablet. If the concentration of the computational particles representing the tablet is below a certain threshold, the particle detaches from the tablet. In this case (as discussed in more detail in Section 2.3.4.), the status of the particle switches from solid to liquid, i.e., from LSM to SPH.

In Figure 1a, a schematic sketch of the model, including the membrane, the colon *haustra* and the fluid inside the colon, is shown.

### 2.3. Colon-Model Geometry

#### 2.3.1. Membrane

**Figure 2:**
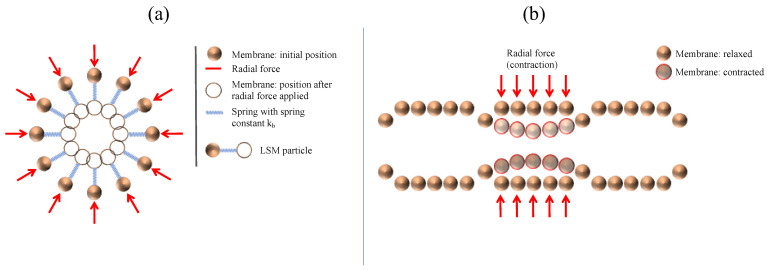
(**a**) 2D representation of the membrane particle anchored by a spring in equilibrium position and after the application of a radial force. (**b**) Illustration the particles representing the colon’s membrane including its characteristic *haustra*, before and after applying an individual radial force to each ring (‘circular muscle’).

The membrane is modelled similarly to [12] by 2500 LSM particles tethered to their initial position with a Hookean spring (Figure 2a). 

An additional Hookean force is applied between the adjacent particles as shown in Figure 1a. The corresponding forces are calculated using Hooke’s law:

(1) $$F_{ij}=k\left(r_{ij}-r_{0}\right),$$

where *F_ij_* is the existing spring force between particle *i* and *j*, *k* is the Hookean constant, *r*_0_ is the equilibrium distance between particle *i* and *j* and *r_ij_* is the current distance. The membrane is composed of 100 rings of 25 particles each. One ring represents one circular muscle fibre of the colon, which can be activated independently to mimic a colonic motor pattern (Figure 2b). 

For activation, we use a radial force applied to the particles of a specific *haustra* to cause contraction or relaxation. Further details of the membrane are shown in Table 1.

#### 2.3.2. Fluid

All models account for the same level of luminal content modelled with 25,758 SPH particles. The amount of fluid is taken from the study of Badley et al. [31] that used scintigraphy to measure the fluid volume in the ascending colon. The average value is 162 mL, with single values ranging from 82 to 303 mL. This corresponds to a filling level of about 40% in the ascending colon [32], which is used in the simulations. In reality, different motility patterns are triggered by different filling levels [14]. However, we fixed the value of 40% for all models to assess the drug release rate of the tablet under different motility patterns for the same level conditions. Resolution analysis (i.e., number of computational particles used to discretize the system) can be found in [12].

#### 2.3.3. Fluid-Structure and Global Boundary Conditions

The SPH equations of motion result from the discrete approximations of the Navier–Stokes equation at a set of points. This set of points results from the discretization of the continuum domain and can be thought of as particles. The particles are characterized particles by their mass, velocity, pressure and density. SPH is based on the mathematical identity:

(2) $$f\left(r\right)=∭f\left(r^{\prime }\right)\delta \left(r-r^{\prime }\right)dr^{\prime }\;\;\;,$$

where *f*(**r**) is any scalar function defined over the volume V and the vector **r** is a three-dimensional point in V. *δ*(**r**) is the three-dimensional delta function which is approximated in the SPH formulations by a smoothing kernel *W* with a characteristic width or smoothing length *h*:

(3) $$\underset{h\to 0}{\lim}W\left(r,h\right)=\delta \left(r\right)$$

In the literature, there are several kernel functions available. In this study, we use the Lucy kernel function [33]. By replacing the delta function by a kernel or smoothing function *W*, the approximation to the function *f*(**r**) results in

(4) $$f\left(r\right)\approx ∭f\left(r^{\prime }\right)W\left(r-r^{\prime },h\right)dr^{\prime }\;\;\;.$$

By discretising over a series of particles of mass $m=\rho \left(r^{\prime }\right)dr^{\prime }$, the identity equation results in

(5) $$f\left(r\right)\approx \underset{i}{\sum }\frac{m_{i}}{\rho_{i}}f\left(r_{i}\right)W\left(r-r_{i},h\right)\;\;\;,$$

where *m_i_* and *ρ_i_* are the mass and density of the *i*th particle, respectively, and *i* ranges over all particles within the smoothing kernel *W* $\left(i.e.,\;\left|r-r_{i}\right|<h\right)$. The discrete approximation of a generic continuous field is represented by Equation (5) and can be used to approximate the Navier–Stokes equation

(6) $$m_{i}\frac{dv_{i}}{dt}=\underset{j}{\sum }m_{i}m_{j}\left(\frac{P_{i}}{\rho_{i}^{2}}+\frac{P_{j}}{\rho_{j}^{2}}+\prod_{i,j}\right)\nabla_{j}W_{i,j}+f_{i}\;\;\;,$$

where *v_i_* is the velocity of particle *i*, *P* is the pressure, *W_i,j_* is the concise form of *W*(**r***_j_–***r***_i_*, *h*), the term ∇*_j_* is the gradient of the kernel with respect to the coordinate r*_j_*, **f***_i_* a volumetric body force, and Π*_i,j_* denotes the viscous forces. There are various expressions for the tensor Π*_i,j_* available in the literature; here we use [34]

(7) $$\Pi_{\mathrm{ij}}=-\alpha h\frac{c_{0}}{\rho_{ij}}\;\frac{v_{ij\;}r_{ij}}{\rho_{ij}^{2}+b\;h^{2}}\;\;,\;$$

where *ρ_ij_* is the density and *v_ij_* the relative velocity of particle *i* and *j*, respectively. α is a dimensionless factor to control the dissipation strength to obtain a stable simulation and *h* is the smoothing length. The constant *b* is introduced and used with *b* ≈ 0.01 to avoid unstable simulations. This is particularly the case with compact particles whose distance between each other is very small. The artificial viscosity can be recognized as an effective kinematic viscosity ν. Depending on the desired effective kinematic viscosity in the simulation, the value of α is chosen accordingly [35]:

(8) $$\nu =\frac{\alpha \;h\;c}{10}$$

The viscosities chosen in this study are consistent to those already used elsewhere [11].

To calculate the pressure forces between the fluid particles and fulfil Equation (6), the Tait equation is used to link the density *ρ* and the pressure *P*

(9) $$P=\frac{c_{0}{}^{2}\;\rho_{0}}{7}\left[{\left(\frac{\rho }{\rho_{0}}\right)}^{7}-1\right],$$

where *c_0_* is the reference speed of sound and *ρ*_0_ the reference density at zero applied stress. Further details of the fluid properties are shown in Table 2.

The solid-fluid interaction is modelled with a soft repulsive potential for the following purposes: to avoid compenetration among the computational solid-fluid particles (i.e., membrane and tablet), to keep the fluid inside the colon and to keep fluid particles out of the solid tablet. The type of soft potential has the following form:

(10) $$E_{ij}=A\left[1+cos\left(\frac{\pi \;r_{ij}}{r_{c}}\right)\right]\;\;\;\;\;\;\mathrm{with}\;\;\;\;\;\;\;r_{ij}<r_{c}$$

Here, *r_ij_* represents the distance between particle *i* and *j*, *r_c_* is the cut-off distance and *A* is an energy constant. The no-slip boundary conditions between the solid and fluid particles are approximated by viscous forces as shown in Equation (7).

#### 2.3.4. Tablet and Tablet Disintegration

In our model, the tablet is composed of 445 particles and is modelled as naturally buoyant. To model the dissolution and disintegration of the tablet, a specific concentration is set for each particle of the tablet, which represents the concentration of the Active Pharmaceutical Ingredient (API) in the tablet. Tablet dissolution is achieved by mass diffusion between the fluid and the tablet particles and between the tablet particles themselves. The diffusive mass balance for multicomponent systems can be written in the SPH framework in the following form [17]:

(11) $$\frac{dw_{i}}{dt}=-\underset{j}{\sum }\frac{m_{i}m_{j}}{\rho_{i}\rho_{j}}\frac{\left(D_{i}+D_{j}\right)\left(C_{i}-C_{j}\right)}{r_{i,j}^{2}}r_{i,j}\cdot \nabla_{j}W_{i,j}\;\;\;,$$

where, *w*_i_ is the mass of the fluid in the particle, *D_i_* is the diffusion coefficient and *C_i_* is the concentration which is associated with each particle *i*. To close Equation (11), *m_i_*, *C_i_* and *ρ_i_* can be linked according to the following relation [17]:

(12) $$w_{i}=C_{i}\frac{m_{i}}{\rho_{i}}$$

A typical ingredient used in colonic formulations is mesalazine [36,37]. Its diffusion coefficient is estimated to be 7.46 × 10^−10^ m^2^s^−1^, determined in an aqueous solution containing triprotic buffer [38], which is very small and requires very long computational times (10 h of real-time, i.e., approx. 10 days of computational time) for observing the complete disintegration of the tablet in the simulation. To reduce the computational time, we initially use a value 10 times higher which only take one hour of real-time (2 days of computational time) for disintegrating the tablet. For more details on the computer architecture used, see Section 2.6.

The solubility of commercially available mesalazine formulations, depending on the pH value, were determined to be 1.2–5.5 mg/mL [10]. This corresponds to a solubility of 50–100% under simulation conditions. Accordingly, the dissolution and disintegration of the tablet are modelled as follows: if the concentration, of at least one of two neighbouring tablet particles falls below *C_B_* = 30% (i.e., 70% dissolved), the bond between these particles is removed, weakening locally the structure of the tablet. When a computational particle has no bonds with any other particle of the tablet, the particle detaches completely simulating the disintegration of the tablet. Finally, when the concentration of the active ingredient in the tablet is below its solubility concentration *C_S_* (defined as 25%, i.e., 75% dissolved), we change the type of the particle from LSM to SPH. For the parameter *C_B_* and *C_S_*, we use generic values as a reference to show the potential of the model. The actual numbers will depend on the physicochemical and mechanical properties of the tablet (i.e., the material used, but also how the tablet is compacted). The values for *C_B_* and *C_S_* should be derived from real data/observations. From the theoretical point of view, the values used for *C_B_* and *C_S_* (i.e., *C_B_* > *C_S_*), provide to the tablet the option to disintegrate (i.e., break into pieces) before it dissolves into the fluid.

In the model, the fluid and the tablet were discretised differently (i.e., different resolution), so that the initial particle distance between the fluid particles and the tablet particles is different. Therefore, a different ‘momentum’ smoothing length is used between the fluid particles and the tablet particles. The ‘diffusion’ smoothing length between the fluid and tablet particles is obtained from a weighted smoothing length based on the smoothing length of the fluid particles and the tablet particles. Further details of the general model parameter and specific model properties are given in Table 1, Table 2, Table 3 and Table 4.

**Figure 3:**
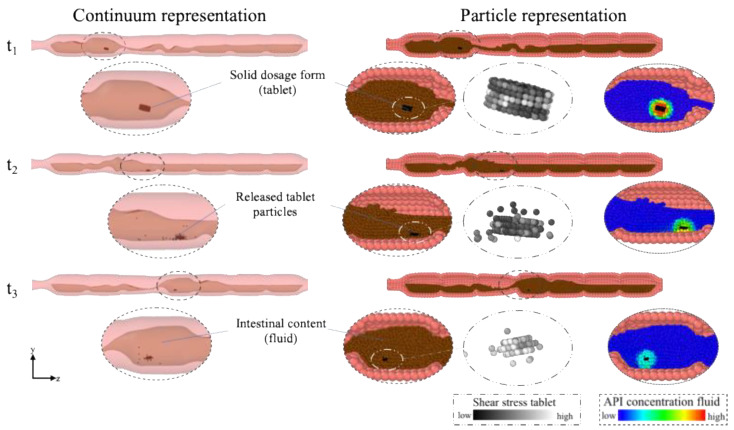
The model is represented in two different forms: ‘Particle representation’ and a more realistic ‘Continuum representation’. This is an example of the dissolution process of a tablet in the colonic environment at three different time steps. Colonic contractions lead to the motion of the fluid and accordingly to the movement of the tablet which dissolves in the intestinal fluid. In the particle representation the shear stress acting on the tablet, and the API concentration in the surrounding of the tablet, i.e., in the fluid is shown.

Figure 3 illustrates an example of the filled colon model, in which a tablet disintegrates and dissolves in the fluid at three different consecutive times *t*. 

The model is represented both in a ‘continuum representation’ and in a ‘particle representation’. The API concentration in the fluid and the tablet are shown in the particle presentation. By diffusion and advection, the API decreases in the tablet and increases in the fluid. The shear stresses present contribute to the tablet breakage into pieces (i.e., disintegration).

Due to the different contractions caused by the motility pattern, the tablet is moved back and forth (see Supplementary Materials Videos S1 and S2) and gradually releases drug particles that dissolve further in the fluid. The mechanism of the disintegration of the tablet presented in this study can be compared to an extended-release (ER) tablet whose coating disintegrates in the upper gastrointestinal and small intestinal environment. When it enters the colon, the ER tablet then behaves similar to an IR tablet and dissolves/disintegrates immediately in the colonic fluid.

Details on the simulation parameter are presented in Table 3. We are aware that under real-world conditions the tablet would not consist of 100% drug, but here the focus is on the different motility pattern and not on the tablet itself. It is also assumed that the tablet/drug is readily soluble and thus the drug release rate is equal to the dissolution rate.

### 2.4. Model Motility Patterns

The motility patterns used in this study are developed from the data produced by [14,39] and analysed in Stamatopoulos et al. [13]. Three different motility patterns at different conditions are considered: baseline (fasted state), stimulated with polyethylene glycol (PEG) electrolyte and stimulated with maltose. The motility patterns differ, for example, in the duration and direction of single contraction waves as well in the frequency of individual contractions. Besides those reported in [13], two additional models are developed to study how much the colonic activity affects the dissolution of the modelled tablet. We use data from [16] to establish a comparison pattern according to the ‘Stimulant PEG’ and the ‘Stimulant Maltose’ patterns, and data from [15] to develop a cyclic propagating pressure wave (CPPW), which is the most frequently motor pattern identified in the colon [15]. The motility patterns consist of different actions: a single contraction, antegrade waves and retrograde waves or a combination of these single actions.

To describe the colonic activity within a specific time interval *t_iv,_*_0_ (e.g., predefined from the total number of available MRI images/analysed time) by a measurable value, Marciani et al. [14] introduced the so-called Motility Index (*MI*). A high *MI* value means high colonic activity, a low *MI* value means, respectively, low activity. The unit of *MI* is ‘segments × s’.

(13) $$MI=\overset{n}{\underset{k=1}{\sum }}{\left(t_{iv}\cdot No_{seg}\right)}_{k},\;\mathrm{with}\;\;n=\frac{t_{iv,0}}{t_{iv}}$$

Here, *t_iv_* is the considered time interval within the total interval *t_iv,_*_0_ and *No_seg_* is the number of segments showing activity during the time interval *t_iv_*. For example, if a Motility Index should be determined for a period of 120 s and the analysed time interval is 20 s, *n* will be equal to 6. Is the colon divided into 4 equally sized segments and only 3 segments show activity in the first time interval, 2 in the second time interval, 4 in the third interval and no activity is recorded during the time intervals 4–6, the Motility Index is thus calculated as follows:

MI=(20·3)+(20·2)+(20·4)=140 segments×s

As mentioned above, the number of sections (*haustra*) varies from model to model, depending on the data available in [13]. Thus, a direct comparison only based on the determined *MI* is not possible. For this reason, we have introduced an additional Specific Motility Index (*SMI*), which also takes the existing number of sections (*haustra*) into account and makes all models comparable.

(14) $$SMI=\frac{\sum_{k=1}^{n}{\left(t_{iv}\cdot No_{seg}\right)}_{k}}{No_{seg,total}},\;\;\;\;\mathrm{with}\;\;n=\frac{t_{iv,0}}{t_{iv}}$$

Here, *No_seg,total_* is the total number of sections (*haustra*) in the specific model. Thus, the result is a value describing the colonic activity per section (*haustra*).

**Figure 4:**
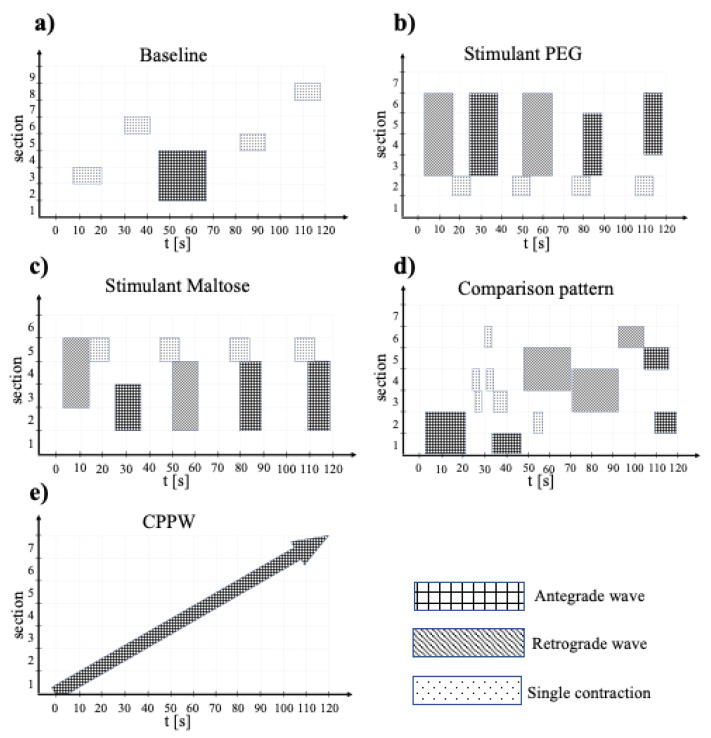
Illustration of the different motility pattern, where (**a**) is the Baseline, (**b**) the Stimulant PEG, (**c**) the Stimulant Maltose, (**d**) the Comparison pattern and (**e**) the CPPW motility pattern. Here, the x-axis represents the time and duration of the actions taking place and the y-axis the colon section (*haustra*) addressed. The different actions are indicated by different hatches.

The data represented in Stamatopoulos et al. [13] are related to a period of two minutes. On this basis, we modelled the different motility patterns for the simulation. The differences between the individual motility patterns are presented in Figure 4a–e. The figure shows the different actions that occur, the duration of each action and the sections that are affected by the action.

For the simulations, these two-minute datasets are repeated accordingly until the desired simulation time is reached (i.e., 5 repetitions for 10 min simulation time). Further details of the motility patterns are provided in Table 5.

**Figure 5:**
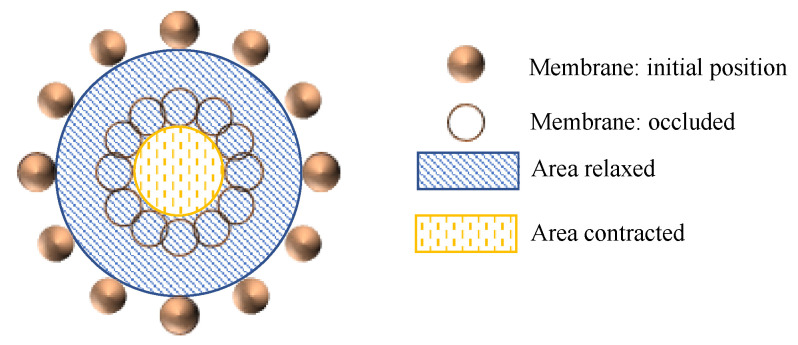
Representation of how the occlusion degree is defined.

The occlusion degree *OD*, as shown in Figure 5 is defined by relating the initial cross-sectional area of the colon, *A_R_* to the cross-sectional area of the colon after contraction, *A_C_* (Equation (15)).

(15) $$OD\;\left[\%\right]=\left(1-\frac{A_{C}}{A_{R}}\right)\cdot 100$$

**Figure 6:**
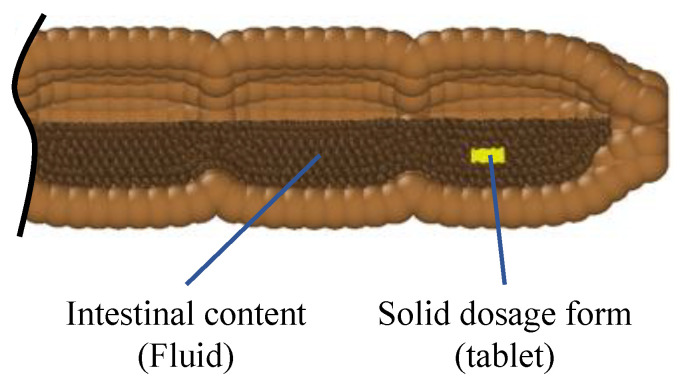
Representation of the initial position of the tablet, which is in all models identical.

At the beginning of the simulation, the tablet is placed in the same initial position in all models (Figure 6). Here, the antegrade direction is from right to left.

### 2.5. Method of Analysis

While the previous section introduced several parameters for characterising the motility patterns (Table 5), this section discusses the parameters used to analyse the numerical results: a measure of the stress on the tablet and the cumulative drug release.

The action of the stresses on the tablet is a crucial factor affecting tablet disintegration. To condensate all the information of the stress tensor in one single parameter, we use the Frobenius norm:

(16) $${\left||\sigma |\right|}_{F}=\sqrt{\overset{3}{\underset{i=1}{\sum }}\overset{3}{\underset{j=1}{\sum }}{\left|\sigma_{ij}\right|}^{2}},$$

where the components *σ_ij_* define the local stress on the *x_i_*, *x_j_* plane.

**Figure 7:**
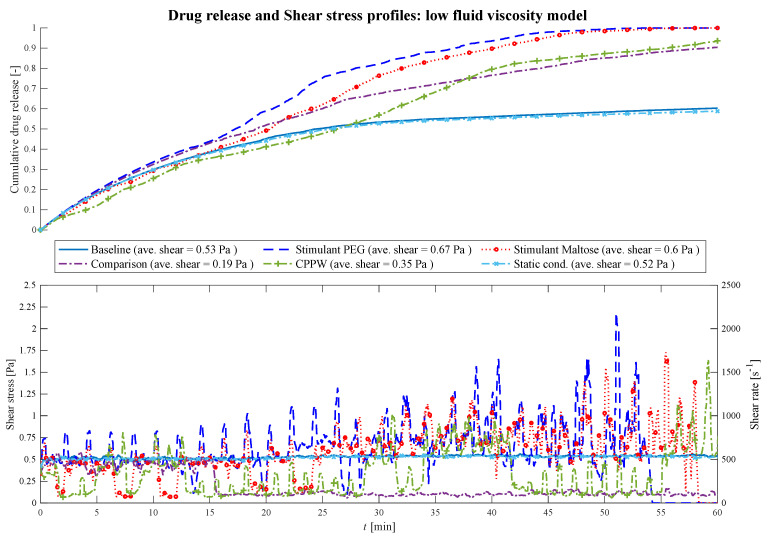
Comparison of the drug release profile of the different models at low fluid viscosity conditions as well as the comparison of the shear stress/shear rate acting on the tablet.

**Figure 8:**
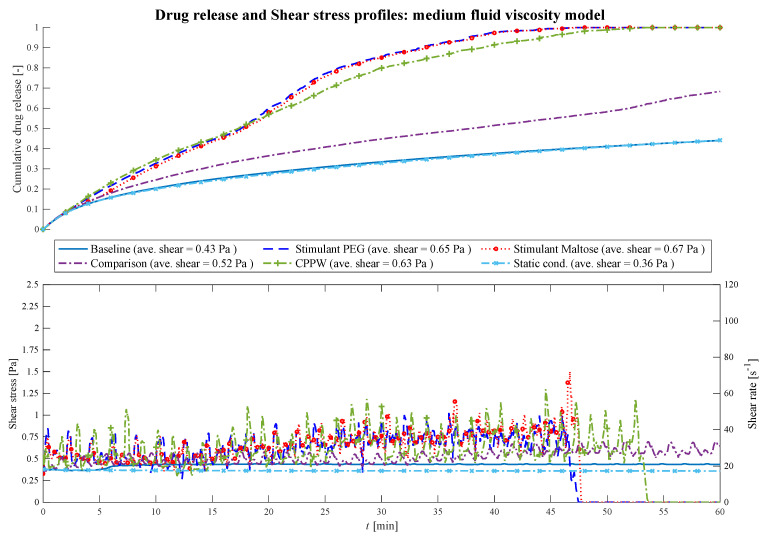
Comparison of the drug release profile of the different models at medium fluid viscosity conditions as well as the comparison of the shear stress/shear rate acting on the tablet.

**Figure 9:**
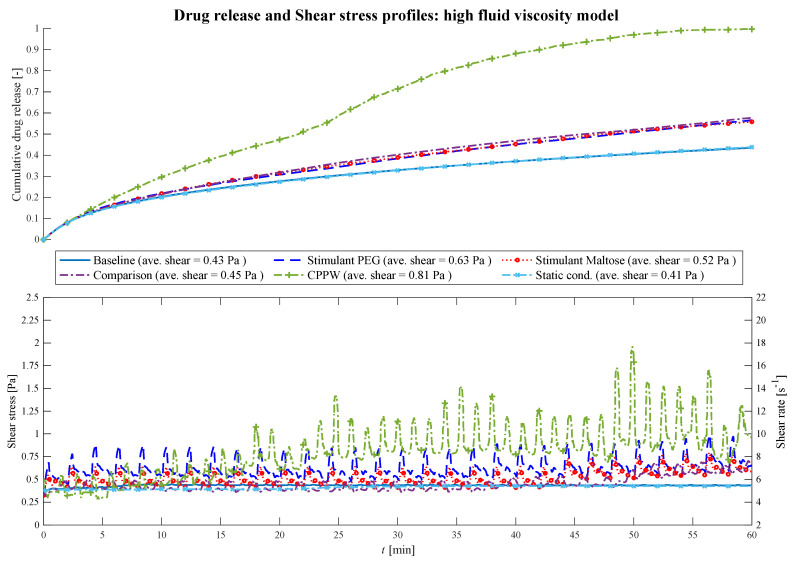
Comparison of the drug release profile of the different models at high fluid viscosity conditions as well as the comparison of the shear stress/shear rate acting on the tablet.

The cumulative drug release (*CDR*) of the tablet (Figure 7, Figure 8, Figure 9 and Figure 13) is calculated according to Equation (17)

(17) $$CDR\;\left[-\right]=1-\frac{\sum c_{i,t}}{\sum c_{0}},$$

where *c*_0_ is the initial tablet particle concentration and *c_i,t_* is the actual concentration of each tablet particle *i* at time *t*.

The shear stress acting on the tablet (Figure 7, Figure 8, Figure 9 and Figure 13) is calculated according to Equation (18)

(18) $$SST\;\left[\mathrm{Pa}\right]=\frac{\sum s_{i,t}}{No_{P,t}},$$

where, *s_i,t_* is the shear stress acting on each tablet particle *i* and *No_P,t_* is the actual number of tablet particles at time *t*.

For the comparison of the different drug release profiles (Figure 7, Figure 8, Figure 9 and Figure 13) the two factor (f_1_ and f_2_) analysis is used [40]. Factor f_1_ is the difference factor in percent, describing the difference (relative error) of two curves at a time point *t*. Factor f_2_ is the similarity factor describing the similarity in the percent drug release between two drug release curves:

(19) $$f_{1}=\frac{\sum_{t=1}^{t=n}\left|R_{t}-T_{t}\right|}{\sum_{t=1}^{t=n}R_{t}}\times 100,$$

(20) $$f_{2}=50\times log\left[\frac{100}{\sqrt{1+\frac{\sum_{t=1}^{t=n}{\left(R_{t}-T_{t}\right)}^{2}}{n}}}\right],$$

where *R_t_* is the drug release for reference formulation at time *t*, *T_t_* is the drug release for comparison formulation at time *t* and *n* the number of time points. Values for f_1_ close to zero (0–15) and f_2_ greater than 50 (50–100) characterise the equivalence of the compared drug release profiles [41,42].

To access the disintegration degree or disintegration time in the case of the complete dissolution of the tablet, the following method is used: Throughout the simulation, the concentration of each tablet particle is tracked. As described in Section 2.2 and Section 2.3.4, each tablet particle is bonded to its neighbour particle until the concentration of one of the neighbouring particles is fallen below a certain threshold concentration and the bond breaks. As soon as no particle with a higher concentration than the threshold concentration is present, then the tablet is completely disintegrated. In the case that the tablet has not completely disintegrated, the degree of disintegration is calculated at *t* = 60 min according to the following equation:

(21) $$\phi \left[\%\right]=\left(1-\frac{No_{T,th}}{No_{T,in}}\right)\cdot 100,$$

where *No_T,th_* is the number of tablet particles with a concentration higher than the threshold concentration and *No_T,in_* is the number of initial tablet particles.

**Figure 10:**
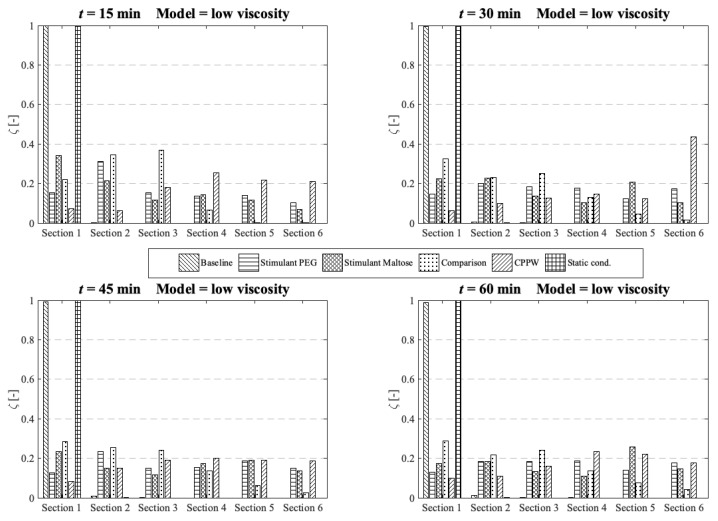
Comparison of the different models in respect to the distribution of the API along the colon at four different times at low fluid viscosity conditions.

**Figure 11:**
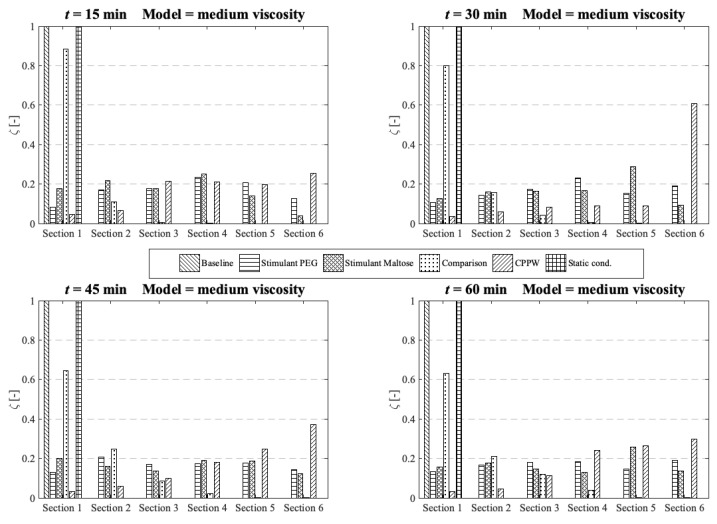
Comparison of the different models in respect to the distribution of the API along the colon at four different times at medium fluid viscosity conditions.

**Figure 12:**
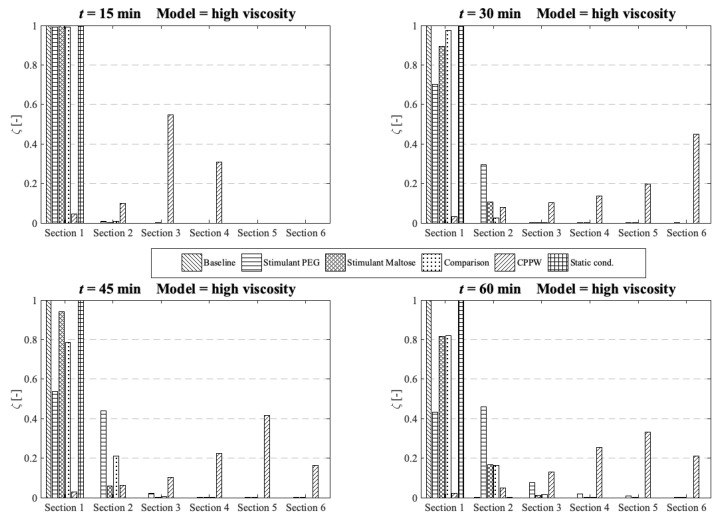
Comparison of the different models in respect to the distribution of the API along the colon at four different times at high fluid viscosity conditions.

The concentration of each tablet particle is tracked throughout the simulation. To assess the performance of each motility pattern on the API distribution along the colon (Figure 10, Figure 11 and Figure 12), we analyse and compare the concentration distribution only of the dissolved API at the corresponding time because only this part is available for absorption. The dimensionless concentration *ζ* in Figure 10, Figure 11 and Figure 12 is calculated according to the following equation:

(22) $$\zeta \;\left[-\right]=\left(\frac{c_{s}}{\sum c_{s}}\right),$$

where *s* is the section number and *c_s_* is the total drug concentration in section *s*.

**Figure 13:**
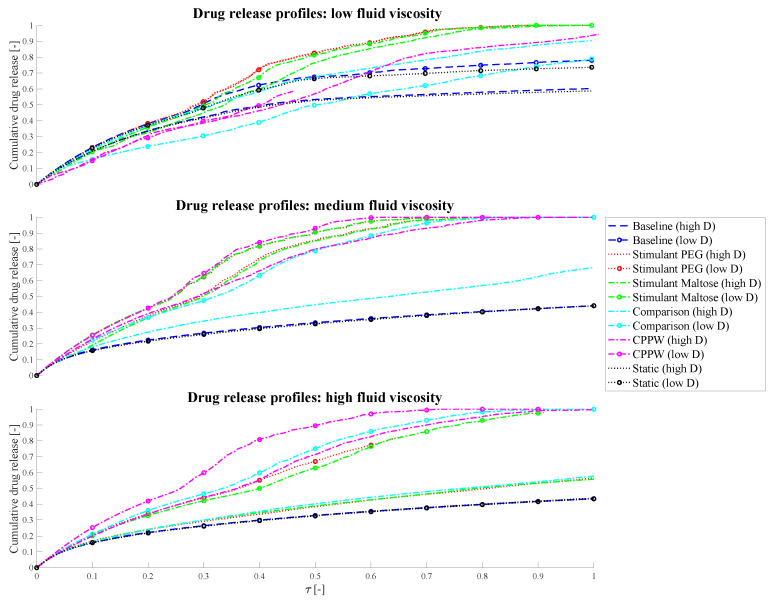
Comparison of the influence of different diffusion coefficients on the drug release profiles, where ‘high D’ represents the drug release profile of the high diffusion coefficient and ‘low D’ the drug release profile of the low diffusion coefficient simulation.

The dimensionless time *τ* (Figure 13) is defined according to the following Equation (23)

(23) $$\tau \;\left[-\right]=\frac{t}{t_{0}}\;,$$

where *t*_0_ is the total simulated time and *t* is the actual time. For the high diffusion data in Figure 13, *t*_0_ = 60 min and for the low diffusion data is *t*_0_ = 600 min. The reason why we can use a reference time *t*_0_ in the definition of *τ* is explained in Appendix A.

### 2.6. Software

In this study, the simulations were performed using the University of Birmingham BlueBEAR HPC service [43], running the simulations on fifteen cores with 60 GB of memory. The open-source code LAMMPS [44,45] is used for the numerical calculations, the open-source code OVITO [46] for the visualisation and MATLAB [47,48] for the postprocessing of the simulation data.

## 3. Results and Discussion

### 3.1. Comparison of Different Motility Pattern on the Drug Release/Disintegration of the Tablet at Different Fluid Viscosities

To assess the influence of the fluid viscosity on the drug release of a tablet in the colon environment, we performed all simulations at three different fluid viscosities: low viscosity (*η_L_* = 1 mPa s), moderate viscosity (*η_L_* = 13 mPa s) and high viscosity (*η_L_* = 98 mPa s). In all three cases, the dissolution process of the tablet, driven by pure diffusion, is represented by a so-called ‘Static’ model. In this model, the membrane does not move at all. Accordingly, no momentum is generated that moves the fluid.

At low fluid viscosity conditions (Figure 7), after a certain time, all motility patterns cause the fluid to move in the colon.

Until *t* = 18 min, in all models, the drug release of the tablet is driven almost by pure diffusion as the drug release profile has the same progression as the ‘Static’ model. The slope of the ‘CPPW’ model is not as steep as in the other models since the tablet is pushed to the surface and, thus, is not in complete contact with the fluid. From *t* = 18 min on, the shear stress becomes a significant factor. In all models, the momentum caused by the different motility pattern is strong enough to set the fluid in motion. Even though the average shear stress does not differ significantly from model to model (see legend Figure 7), recurring shear stress peaks enhanced drug release. Additionally, small fluid motions, such as those that occur in the ‘Baseline’ model, lead to somewhat higher advection-induced mass transfer and thus to a higher release rate of the tablet compared to the ‘Static’ model. In a low viscous fluid environment, a complete dissolution of a tablet is only achieved, in the case of the ‘Stimulant PEG’, ‘Stimulant Maltose’ motility pattern. Figure 7 shows how the shear stress acting on the tablet increases with time as the wall motion transfers more and more momentum to the fluid. In the case of the ‘Comparison’ pattern, the tablet is pushed back and forth until *t* = 15 min, where the tablet is pushed to the end next to the initial position of the tablet, where the tablet remains until the end of the simulation. Here, the tablet does not receive significant momentum from the contractions, but some fluid still flows around it which leads to an increased advection-induced mass transfer and thus to a higher drug release. The sparse shear stress peaks in the ‘CPPW’ model are observed because only one wave is travelling from one end of the colon to the other, and always in one direction. Thus, at low fluid viscosity condition, the momentum induced by the wave reaches the tablet only occasionally.

When comparing the three different motility patterns found in [13,14,39], at low fluid viscosity conditions, it can be seen from Figure 7 that the drug release profile of the ‘Stimulant PEG’ and ‘Stimulant Maltose’ model do not show significant differences (i.e., f_1_ = 12.0%, f_2_ = 57.7%). The drug release rate of the ‘Stimulant PEG’ model is somewhat higher than the ‘Stimulant Maltose’ model where the drug is completely released from the tablet at *t* = 54 min and *t* = 59 min, respectively. The release rate in the ‘Baseline’ model is much smaller than in the other two models and does not deviate much from the ‘Static’ model (i.e., f_1_ = 1.6%, f_2_ = 93.9%).

At higher viscosities (Figure 8) the fluctuations of the shear stress decrease and the drug release profiles become similar (i.e., PEG—Maltose: f_1_ = 2.5%, f_2_ = 87.9%; PEG—CPPW: f_1_ = 6.2%, f_2_ = 68.5%; Maltose—CPPW: f_1_ = 6.8%, f_2_ = 69.5%; Static—Baseline: f_1_ = 1.3%, f_2_ = 97.8%). 

The most pronounced effect is in the ‘Comparison’ model where the drug release of the tablet decreases significantly compared to the low viscosity model. Only the momentum generated from the motility patterns of the models ‘Stimulant PEG’, ‘Stimulant Maltose’ and ‘CPPW’ are strong enough to agitate the fluid sufficiently with regard to an increased advection and thus accelerated drug release rate. In all three models, the increased drug release rate leads to complete disintegration of the tablet within the simulation time (*t* = 54 min). The most significant effect can be seen in the case of the ‘CPPW’. At higher viscosity, the tablet is not pushed to the surface of the fluid and remains in the fluid for the majority of the time (see Supplementary Materials Videos S3 and S4).

The comparison of the ‘Stimulant PEG’, ‘Stimulant Maltose’ and ‘Baseline’ cases at moderate fluid viscosities shows that the ‘Stimulant PEG’ and ‘Stimulant Maltose’ model has almost the same drug release profile. In both models, the drug of the tablet is completely released at *t* = 48 min. Since in the ‘Baseline’ model the impulse induced by the contraction is not sufficient to move the fluid, the drug release profile shows the same progression as the ‘Static’ model.

In the case of the highest fluid viscosity used in this study (Figure 9), the motility pattern of the ‘CPPW’ model is the only pattern capable of agitating the fluid at a high level, generating sufficient shear stress to promote the drug release process and lead to almost complete drug release of the tablet. 

The contractions performed in the ‘Baseline’ model do not influence the drug release process. The impulse they generate is too weak to induce any influential fluid motion. At high viscosity conditions, the motility patterns in the ‘Stimulant PEG’, ‘Stimulant Maltose’ and ‘Comparison’ models result in almost the same drug release profile (i.e., Comparison—PEG: f_1_ = 3.1%, f_2_ = 89.1%; Comparison—Maltose: f_1_ = 2.0%, f_2_ = 95.1%; PEG—Maltose: f_1_ = 1.1%, f_2_ = 97.5%), even though they show partly different drug release profiles at lower viscosities.

The average shear stresses for each model and each viscosity are given in Figure 7, Figure 8 and Figure 9. Across all models and viscosities, we found 120 Pa for the maximum local shear stress acting on the surface of the tablet. These values correspond very well with the shear stresses found in other studies, even though they focused on the stomach [49,50].

The shear rates acting on the tablet fluctuate sharply between 100 and 2170 s^−1^ at low fluid viscosity, between 18 and 70 s^−1^ at medium viscosity and between 5.5 and 18 s^−1^ at high fluid viscosity. In the USP II, the shear rates are constant for a given location in the vessel and increase proportionally to the paddle speed [51]. The shear rates found in the USP II at fluid conditions comparable to the ‘low viscosity model’ are in the range of 5 s^−1^ at 25 rpm and 250 s^−1^ at 200 rpm paddle speed [51,52,53,54]. The linear shear rate profile of the USP II is not characteristic of the colonic environment, where the motility pattern evokes sharp shear rate spikes that significantly affect the dissolution/disintegration process. Especially at low fluid viscosity, the hydrodynamic parameters (i.e., shear rate and fluid velocity) enhance the dissolution/disintegration rate.

In addition to the dissolution profile, the degree of disintegration or the disintegration time for the complete disintegration of the solid dosage form (tablet) are also important parameters influencing the drug release rate. The corresponding results of all motility patterns/models and all fluid viscosities are summarised in Table 6.

### 3.2. Comparison of the API Distribution along the Colon

Standard dissolution/drug release profiles, as commonly performed to access the properties of a solid dosage form, do not give any valuable information about the concentration gradient of the API along the colon. This information is important to determine the efficacy of the API in terms of drug absorption. To gain more insight into the concentration gradient along the colon, we divided the colon into six equal sections to see how the API concentration is distributed over time. Here, section one includes the initial position of the tablet and section six is at the end of the colon. The comparison and analysis of the concentration in each section and model are carried out at four different time points and the three different fluid viscosities: low viscosity, medium viscosity and high velocity.

At low fluid viscosity (Figure 10) the models: ‘Stimulant PEG’, ‘Stimulant Maltose’, ‘Comparison’ and the ‘CPPW’ model can distribute the API completely along the whole colon at *t* = 60 min, but only the ‘Stimulant PEG’, ‘Stimulant Maltose’ and ‘CPPW’ models show a very uniform API distribution. 

These are also the models which achieved the highest drug release from the tablet (Figure 7). The ‘Comparison’ model is not as effective as the other three models in terms of API distribution, which means that in this model only a small part of the API reaches the last section at the end of the colon.

Since the wave in the ‘CPPW’ model only propagates in one direction (antegrade), the tablet is pushed to the end of the colon and dissolves there. Thus, the highest concentration in the course of the simulation (e.g., *t* = 30 min) is found at the end of the model. A backflow caused by the wave prevents the accumulation of the API at the end of the colon.

The ‘Baseline’ model is only able to transfer parts of the dissolved API into the sections one to four whereby the largest amount of dissolved API remains in the first segment.

At medium fluid viscosity conditions (Figure 11), at *t* = 60 min, only the ‘Stimulant PEG’, ‘Stimulant Maltose’ and ‘CPPW’ models distribute a notable portion of API along the entire colon. 

In terms of uniform distribution of API across all segments, only the ‘Stimulant PEG’ and ‘Stimulant Maltose’ model show good results. As already seen at low viscosity conditions, in the ‘CPPW’ model the tablet is captured by the wave and pushed to the end of the colon where it dissolves. As the reflux is less pronounced at higher fluid viscosity conditions, at *t* = 60 min, the API accumulates in the last three sections of the colon. Nevertheless, the reflux generated in this model influences the mixing of the intestinal contents. By extending the simulation time, the reflux would very likely lead to an even API distribution along the colon. At the end of the simulation time, the ‘Comparison’ model is capable to distribute a notable amount of dissolved API across the first four segments, whereas the largest fraction remains in the first segment. The ‘Baseline’ model is not even capable to move a fraction of the dissolved API in the neighbouring segment.

In the case of high fluid viscosity conditions (Figure 12), only the motility pattern of the ‘CPPW’ model can distribute a significant amount of dissolved API across all segments.

As expected, the motility pattern of the ‘Baseline’ model does not distribute the dissolved API. The ‘Comparison’ model moves some dissolved API in segment two to four, but a notable amount is only found in segments one to three. The ‘Stimulant Maltose’ model is comparable with the ‘Comparison’ model with the small difference that the ‘Stimulant Maltose’ model also distributes a very small amount of API into segments five and six. The relative amount of API in sections one to three is in both models almost the same. The ‘Stimulant PEG’ model can move a significant amount of dissolved API into the second segment and still a small but noteworthy amount into the third and fourth segment. The API amount in segment five and six is very small, but still, a very small amount reaches these segments.

The results show that the effectiveness of the motility pattern in terms of API distribution along the colon is highly dependent on the viscosity of the intestinal content. Contrary to the assumption that the motility pattern with the highest average shear stress automatically indicate the fastest drug release rate, the motility pattern that provokes frequent single shear stress peaks seem to be more effective in achieving a higher drug release rate. Another important parameter in terms of tablet drug release is the position of the tablet and the motility pattern itself. To achieve a faster tablet drug release compared to pure diffusion (‘Static’ model), the tablet must be in a region in which it can be caught by the motility pattern. When we compare the three different motility patterns found in [13,14,39] in terms of their performance in drug release and drug distribution, the parameters described above play a crucial role. The motility pattern shows different performances in terms of tablet drug release and also in terms of the distribution of the dissolved drug. The motility pattern of the ‘Baseline’ model is extensively ineffective compared to the other two motility pattern found. The motility patterns of the ‘Stimulant PEG’ and ‘Stimulant Maltose’, on the other hand, show very similar performances in terms of tablet drug release. The motility pattern ‘Stimulant PEG’ seems to be marginally more efficient in terms of drug distribution along the colon at higher viscosities.

The ‘Baseline’ motility pattern is the most predominant environment in a healthy human. As shown in the results, at higher fluid viscosities, the ‘Baseline’ motility pattern is not able to induce any influential fluid motion that would significantly affect the disintegration/drug release process. This biorelevant knowledge cannot be easily assessed with compendial disintegration/dissolution apparatuses which makes the in silico model valuable. From the results, it can deduce that care should be taken for the formulation design to mitigate prolong and/or partial disintegration/drug release.

### 3.3. Influence of the Diffusion Coefficient on the Drug Release from Tablet

As mentioned above, the required computational time is significantly higher when a lower diffusion coefficient is used, and complete drug release of the tablet is aimed for—at least for some motility patterns. Additionally, the tablet should dissolve in about one hour, which is not achievable with a low diffusion coefficient.

To estimate the impact of diffusion coefficients that differs by one order of magnitude on the drug release process in the models used in this study, we performed a dimensional analysis of the system and additionally ran simulations of each model for 10 days, regardless of how much time can be simulated in that period—this also varies from model to model. For this reason, the drug release profiles/results represented in Figure 13 may not show results for the entire time axis.

However, the target simulation time was 10 h (i.e., also one order of magnitude higher than in the case of high diffusion coefficient used). Results from the dimensional analysis confirm that it is possible to compare disintegration/dissolution profiles between different diffusion coefficients by proportionally rescaling time. Further detail of the analysis can be found in Appendix A. From the dimensional analysis, it can be obtained that the differences of the curves shown in Figure 13 are resulting from the fluid dynamics in the colon as the diffusion coefficient and time are scaled proportionally.

The different models (motility pattern) and different diffusion coefficients are compared based on dimensionless time (Equation (22)).

In the simplest case, which is the ‘Static’ model, the drug release profiles coincide at medium and high fluid viscosities very well, which should be the case as the diffusion and time are proportional. In the case of low fluid viscosity, the drug release profiles are slightly different but show almost parallel curves. In this case, even if there is no fluid movement, the tablet can move very slightly, especially when it releases drug particles and the size of the tablet changes. These very small movements can result in a very small amount of advection in addition to diffusion and cause the difference in drug release profiles. As soon as a fluid motion occurs, introduced by the different motility pattern, the driving parameter in terms of drug release is very much dependent on the position of the tablet and on how the accelerated fluid can reach the tablet. At low fluid viscosity conditions, the tablet tends to be pushed to the surface of the fluid which slows down the drug release rate and consequently the dissolution rate. This phenomenon can especially be recognised in the ‘Comparison’ model, where a higher drug release can be achieved at higher diffusion coefficient conditions. In the case of a lower diffusion coefficient but longer simulation time, the worst case with regard to drug release has occurred. The tablet is pushed to the surface of the fluid and additionally into a region where the fluid circulation is quite low. Even the fact that significantly more fluid movement can be achieved in 10 h compared to 1 h, and thus the drug release rate should be higher with lower diffusion coefficients, is not given in this case. Here, diffusion is the driving parameter. The increased proportion of advection, due to prolonged fluid movement, in addition to pure diffusion is particularly well seen in the models with higher fluid viscosities. Here, in all cases, a higher or/and faster relative drug release could be achieved with lower diffusion and longer simulation time compared to the case of the higher diffusion coefficient and shorter simulation time.

### 3.4. Strengths and Limitations of the Model

The strengths of the model include that the anatomy, fluid volumes and motility patterns are informed by robust clinical data. The model presented within this paper is capable of simultaneously capturing data on drug dissolution and distribution within the ascending colon under a range of motility patterns and fluid viscosities. Generation of such data in vivo is complex due to the relative inaccessibility of the ascending colon plus the complexity in controlling either fluid viscosity or motility. However, it is recognised that validation of the model against clinical data would offer great benefits in demonstrating the utility of the model.

In the absence of clinical data that directly replicates observations in the model correlations have been sought from the most relevant literature to demonstrate the utility of the developed model. A comparison of 5-ASA pharmacokinetics in healthy adults; adults with CD and adults with UC showed that the time to reach the colon was faster with greater overall exposure for the diseased patients compared to the healthy controls [55]. This increased exposure is likely to be due to a combination of factors: an increase in permeability due to the inflamed mucosa or the reduction in viscosity of the colonic fluids in patients with CD or UC or the greater frequency of propagating contractions in the colon [56,57]. The impact of reduced viscosity and greater frequency of contractions provides consistency with our model. The regional colonic distribution of material has been shown to differ between healthy adults and those with UC where those with UC had a significantly lower percentage in the left side of the colon compared to controls [58]. The rapid transit observed in UC as a result of greater motility would explain these data, which is consistent with the findings from our model.

The rapid distribution of material within the ascending colon was demonstrated in a paper using scintigraphy to visualise mesalamine microspheres where complete distribution was observed within 30 min of entry to the ascending colon [59] which is consistent with the low viscosity scenario presented within our model.

Thorpe et al. [60], using a dynamic model of colonic concentrations that mimics published transit time data, reported that the distribution of 5-ASA within the colon was shown to change in response to a change in motility patterns with reduced motility resulting in higher concentrations of 5-ASA [60] which is also consistent with data from our model. This work considered a simple immediate release formulation as a first step in the development of the model. Future work will include evaluation of alternative formulations that target the colon, particularly formulations where clinical data is available so that the output can be compared to the existing clinical data.

## 4. Conclusions

This study describes the development of a computational model to describe the drug release from and the disintegration of a solid dosage form (tablet) and the distribution of the API in the environment of the ascending colon. The model considers different motility patterns as well as different fluid viscosities.

The relationships between fluid viscosity, motility pattern, and consequently tablet drug release/disintegration and distribution along the ascending colon are investigated. For the targeted drug delivery predictions, conventional in vitro dissolution tests are commonly performed under standardised conditions and limited abilities to mimic the real colonic conditions. In particular, this applies to the different motility patterns that occur in the colonic environment [9]. Our results show how the combination of different motility patterns and fluid viscosities exerts different shear stresses on the tablet, resulting in different drug release rates and different drug distributions along the colon. Compared to the standard drug dissolution tests and apparatuses currently in use [9], our model not only provides a more realistic environment but also an enhanced insight into the dissolution/drug release process itself, that to the best of our knowledge, represents the first step towards the ability to create spatiotemporal tablet drug release profiles. Since it can replicate almost any motility pattern, including propagating distance, propagating velocity, propagating direction or even single contractions and different occlusion degrees, the model allows us to assess how different motility patterns affect the dissolution process of a solid dosage form. Additionally, of interest is the inter-individual variability, and this model can predict (to some extent) the variability for a given dosage form in a range of people. From the results, we can conclude that the movement of the fluid, caused by the different motility patterns, is one of the most important parameters in terms of drug release, besides diffusion, and the most important parameter when the tablet is exposed to the fluid flow. The model provides further insight into whether the motility pattern can transport the drug in the desired concentration to the sites to be treated. The results obtained can be of great importance for both medical research and pharmaceutical applications, especially for the design and optimisation of a modified release dosage form.

## Figures and Tables

**Table 1:** Model parameter of the Membrane.

Parameter Membrane	Value
**SPH**	
Number of SPH particles (1 layer)	2500
Mass of each particle *m_M,_*_0_	3.89 × 10^−4^ kg
**LSM**	
Hookean coefficient (bonds) *k_M,b_*	0.2 J m^−2^
Hookean coefficient (position anchor) *k_M,p_*	0.012 J m^−2^
Viscous damping coefficient *k_M,v_*	1.0 × 10^−2^ kg s^−1^
Equilibrium distance *r_M,_*_0_	6.28 × 10^−3^ m

**Table 2:** Model parameter of the Fluid.

SPH Parameter Fluid	Value
Number of SPH fluid particles	25,758
Mass of each particle *m_F,low viscosity_*	1.19 × 10^−5^ kg
Mass of each particle *m_F,medium viscosity_*	1.21 × 10^−5^ kg
Mass of each particle *m_F,high viscosity_*	1.22 × 10^−5^ kg
Density (fluid) *ρ_F,low viscosity_*	1000 kg m^−3^
Density (fluid) *ρ_F,medium viscosity_*	1017 kg m^−3^
Density (fluid) *ρ_F,high viscosity_*	1020 kg m^−3^
Dynamic viscosity (fluid) *η_L,low viscosity_*	1 mPa s
Dynamic viscosity (fluid) *η_L,medium viscosity_*	13 mPa s
Dynamic viscosity (fluid) *η_L,high viscosity_*	98 mPa s

**Table 3:** Fundamental model parameter.

Parameter	Value
**SPH**	
Artificial speed of sound *c*_0_	0.1 m s^−1^
Time-step Δ*t*	5.0 × 10^−4^ s
Smoothing length membrane *h*	9.42 × 10^−3^ m
Momentum–Smoothing length (fluid) *h_M,F_*	4.71 × 10^−3^ m
Momentum–Smoothing length (tablet) *h_M,T_*	1.41 × 10^−3^ m
Momentum–Smoothing length (fluid/tablet) *h_M,F/T_*	4.71 × 10^−3^ m
Diffusion–Smoothing length (fluid/tablet) *h_D,F/T_*	3.35 × 10^−3^ m
Diffusion–Smoothing length (fluid) *h_D,F_*	4.71 × 10^−3^ m
Diffusion–Smoothing length (tablet) *h_D,T_*	1.41 × 10^−3^ m
Diffusion coefficient (tablet) *D_T_*	1.0 × 10^−30^ m^2^s^−1^
Diffusion coefficient (fluid/tablet) *D_F/T_*	7.46 × 10^−9^ m^2^s^−1^

**Table 4:** Model parameter of the Tablet.

Parameter Tablet	Value
**SPH**	
Number of SPH tablet particles	445
Mass of each particle *m_T,low viscosity_*	8.82 × 10^−7^ kg
Mass of each particle *m_T,medium viscosity_*	8.97 × 10^−7^ kg
Mass of each particle *m_T, high viscosity_*	9.00 × 10^−7^ kg
Density (Tablet) ρ*_T,low viscosity_*	1000 kg m^−3^
Density (Tablet) ρ*_T,medium viscosity_*	1017 kg m^−3^
Density (Tablet) ρ*_T,high viscosity_*	1020 kg m^−3^
**LSM**	
Hookean coefficient *k_T,b_*	0.2 J m^−2^
Equilibrium distance (linear bonds) *r_TL,_*_0_	0.012 J m^−2^
Equilibrium distance (diagonal bonds) *r_TD,_*_0_	6.28 × 10^−3^ m

**Table 5:** Model parameter motility patterns.

Parameter	Baseline	PEG	Maltose	Comparison	CPPW
No. of sections	9	7	6	7	7
Motility index [segment × s]	140	460	380	300	280
Specific motility index [segment × s × s_total_^−1^]	16	66	63	43	40
Occlusion velocity ‘wave’ [cm/s]	0.1	2.3	2.3	0.8	0.1
Occlusion velocity ‘single contraction’ [cm/s]	0.1	0.1	0.1	0.25	-
Wave travel velocity [cm/s]	0.9	2.5	2.5	0.85	1.0
Occlusion degree *OD* [%]					
Single contraction 1	25	60	60	30	-
Single contraction 2	25	60	60	50	-
Single contraction 3	25	60	60	60	-
Single contraction 4	25	60	60	40	-
Single contraction 5	-	-	-	50	-
Single contraction 6	-	-	-	30	-
Antegrade wave 1	20	75	75	40	-
Antegrade wave 2	-	55	55	55	40
Antegrade wave 3	-	40	40	75	-
Antegrade wave 4	-	-	-	40	-
Retrograde wave 1	-	75	75	40	-
Retrograde wave 2	-	40	40	75	-
Retrograde wave 3	-	-	-	40	-

**Table 6:** Disintegration degree or disintegration time of the solid dosage form (tablet) for all models/motility patterns at different fluid viscosities: % = degree of tablet disintegration *ϕ* at t = 60 min in %; min = time in minutes until complete tablet disintegration.

Model/Motility Pattern	Low Viscosity	Medium Viscosity	High Viscosity
Static	15%	0%	0%
Baseline	16%	0%	0%
PEG	54.3 min	45.6 min	16%
Maltose	58.4 min	46.1 min	13%
Comparison	81%	47%	33%
CPPW	93%	53.7 min	57.8 min

**Figure A1:**
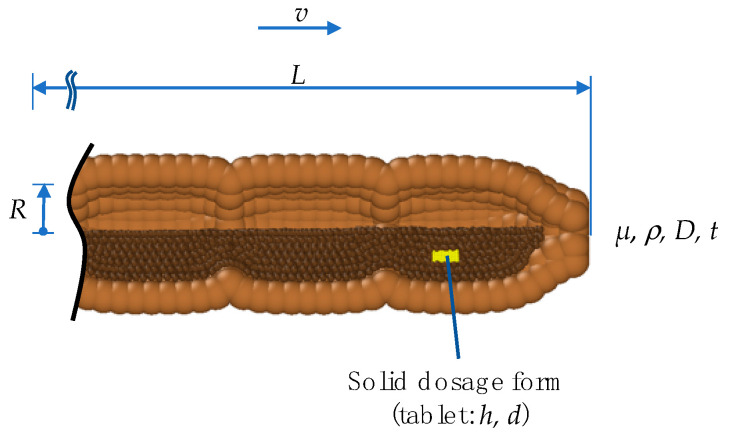
System parameter for the Dimensional Analysis.

## Data Availability

The code used for the simulations is freely available on request from the corresponding author.

## Supplementary Materials

The following are available online at https://www.mdpi.com/article/10.3390/pharmaceutics13060859/s1, Video S1: Example 1, how the tablet moves back and forth in the colonic content (section from the simulation ‘Stimulant PEG’ at medium fluid viscosity), Video S2: Example 2, how the tablet moves back and forth in the colonic content (section from the simulation ‘Stimulant Maltose’ at medium fluid viscosity), Video S3: Section from the simulation ‘CPPW’ at low fluid viscosity, where the tablet is pushed to the surface of the fluid, Video S4: Section from the simulation ‘CPPW’ at medium fluid viscosity, where the tablet remains in the fluid.

## Appendix A

According to the Buckingham π theorem, a physically meaningful equation involving *n* of physical variables can be rewritten in terms of a set of *p* = *n* − *k* dimensionless parameters *Π*_1_, *Π*_2_, …, *Π_p_*_,_ where *k* is the number of physical dimensions involved.

In the case under investigation, we can ideally express the results as mathematical function *f* of the type

(A1) t=f(v,h,d,μ,ρ,D,R,L)   ,

where all the variables and their respective physical units are reported in Table A1 and shown in Figure A1. Since we have 9 variables and 3 units, we can rewrite Equation (A1) based on 6 dimensionless parameters,

(A2) $$\Pi_{1}=\varphi \left(\Pi_{2},\ldots ,\Pi_{6}\right)\;\;\;,$$

**Table A1:** Variables for the Dimensional Analysis.

	Variable	Unit	Description
(1)	*t*	s	dissolution time
(2)	*v*	m s^−1^	velocity of wave
(3)	*h*	m	thickness of tablet
(4)	*d*	m	diameter of tablet
(5)	*µ*	kg m^−1^ s^−1^	dynamic viscosity
(6)	*ρ*	kg m^−3^	density
(7)	*D*	m^2^ s^−1^	diffusion coefficient
(8)	*R*	m	radius of colon
(9)	*L*	m	length of colon

Table A2 shows a possible way to combine the nine dimensional variables into six dimensionless parameters. As we can see, fixed the radius *R*, the dimensionless time *Π_1_* is proportional to the diffusivity, which justify the use of

$$t_{0}\propto \frac{R^{2}}{D}$$

in Equation (23).

**Table A2:** Dimensionless variables for the Dimensional Analysis.

$\Pi_{1}\propto \frac{t\;D}{R^{2}}$	$\Pi_{2}\propto \frac{R}{L}$	$\Pi_{3}\propto \frac{d}{h}$
$\Pi_{4}\propto \frac{d}{L}$	$\Pi_{5}\propto \frac{R\;v}{D}$	$\Pi_{6}\propto \frac{\rho \;v\;R}{µ}$

## References

[1] Alatab S., Sepanlou S.G., Ikuta K., Vahedi H., Bisignano C., Safiri S., Sadeghi A., Nixon M.R., Abdoli A., Abolhassani H. (2020). The global, regional, and national burden of inflammatory bowel disease in 195 countries and territories, 1990–2017: A systematic analysis for the Global Burden of Disease Study 2017. Lancet Gastroenterol. Hepatol..

[2] Goffredo R., Pecora A., Maiolo L., Ferrone A., Guglielmelli E., Accoto D. (2016). A swallowable smart pill for local drug delivery. J. Microelectromech. Syst..

[3] Teruel A.H., Gonzalez-Alvarez I., Bermejo M., Merino V., Marcos M.D., Sancenon F., Gonzalez-Alvarez M., Martinez-Manez R. (2020). New insights of oral colonic drug delivery systems for inflammatory bowel disease therapy. Int. J. Mol. Sci..

[4] Amidon S., Brown J.E., Dave V.S. (2015). Colon-targeted oral drug delivery systems: Design trends and approaches. AAPS PharmSciTech.

[5] Christensen J., Johnson L.R. (1994). Physiology of the Gastrointestinal Tract.

[6] Kumar S.P., Prathibha D., Parthibarajan R., Reichal C.R. (2012). Novel colon specific drug delivery system: A review. Int. J. Pharm. Pharmaceut. Sci..

[7] Murray K., Hoad C.L., Mudie D.M., Wright J., Heissam K., Abrehart N., Pritchard S.E., Al Atwah S., Gowland P.A., Garnett M.C. (2017). Magnetic resonance imaging quantification of fasted state colonic liquid pockets in healthy humans. Mol. Pharm..

[8] Watts P.J., Illum L. (1997). Colonic drug delivery. Drug Dev. Ind. Pharm..

[9] Long M., Chen Y., Qiu Y., Chen Y., Zhang G.G.Z., Liu L., Porter W.R. (2009). Developing Solid Oral Dosage Forms.

[10] Tenjarla S. (2015). Dissolution of commercially available mesalamine formulations at various pH levels. Drugs RD.

[11] Stamatopoulos K., Batchelor H.K., Simmons M.J.H. (2016). Dissolution profile of theophylline modified release tablets, using a biorelevant Dynamic Colon Model (DCM). Eur. J. Pharm. Biopharm..

[12] Schütt M., Stamatopoulos K., Simmons M.J.H., Batchelor H.K., Alexiadis A. (2020). Modelling and simulation of the hydrodynamics and mixing profiles in the human proximal colon using Discrete Multiphysics. Comput. Biol. Med..

[13] Stamatopoulos K., Karandikar S., Goldstein M., O’Farrell C., Marciani L., Sulaiman S., Hoad C.L., Simmons M.J.H., Batchelor H.K. (2020). Dynamic colon model (DCM): A cine-MRI informed biorelevant in vitro model of the human proximal large intestine characterized by positron imaging techniques. Pharmaceutics.

[14] Marciani L., Garsed K.C., Hoad C.L., Fields A., Fordham I., Pritchard S.E., Placidi E., Murray K., Chaddock G., Costigan C. (2014). Stimulation of colonic motility by oral PEG electrolyte bowel preparation assessed by MRI: Comparison of split vs single dose. Neurogastroenterol. Motil..

[15] Dinning P.G., Wiklendt L., Maslen L., Gibbins I., Patton V., Arkwright J.W., Lubowski D.Z., O’Grady G., Bampton P.A., Brookes S.J. (2014). Quantification of in vivo colonic motor patterns in healthy humans before and after a meal revealed by high-resolution fiber-optic manometry. Neurogastroenterol. Motil..

[16] Sarna S.K. (2010). Colonic Motility: From Bench Side to Bedside.

[17] Alexiadis A. (2015). The discrete multi-hybrid system for the simulation of solid-liquid flows. PLoS ONE.

[18] Alexiadis A. (2015). A new framework for modelling the dynamics and the breakage of capsules, vesicles and cells in fluid flow. Procedia IUTAM.

[19] Alexiadis A., Stamatopoulos K., Wen W., Batchelor H.K., Bakalis S., Barigou M., Simmons M.J. (2017). Using discrete multi-physics for detailed exploration of hydrodynamics in an in vitro colon system. Comput. Biol. Med..

[20] Ariane M., Allouche M.H., Bussone M., Giacosa F., Bernard F., Barigou M., Alexiadis A. (2017). Discrete multi-physics: A mesh-free model of blood flow in flexible biological valve including solid aggregate formation. PLoS ONE.

[21] Ariane M., Kassinos S., Velaga S., Alexiadis A. (2018). Discrete multi-physics simulations of diffusive and convective mass transfer in boundary layers containing motile cilia in lungs. Comput. Biol. Med..

[22] Ariane M., Wen W., Vigolo D., Brill A., Nash F.G.B., Barigou M., Alexiadis A. (2017). Modelling and simulation of flow and agglomeration in deep veins valves using discrete multi physics. Comput. Biol. Med..

[23] Mohammed A.M., Ariane M., Alexiadis A. (2020). Using discrete multiphysics modelling to assess the effect of calcification on hemodynamic and mechanical deformation of aortic valve. ChemEngineering.

[24] Liu G.R., Liu M.B. (2003). Smoothed Particle Hydrodynamics: A Meshfree Particle Method.

[25] Kot M., Nagahashi H., Szymczak P. (2015). Elastic moduli of simple mass spring models. Vis. Comput..

[26] Lloyd B.A., Szekely G., Harders M. (2007). Identification of spring parameters for deformable object simulation. IEEE Trans. Visualizat. Comput. Graph..

[27] Pazdniakou A., Adler P.M. (2012). Lattice spring models. Trans. Porous Media.

[28] Rahmat A., Barigou M., Alexiadis A. (2020). Numerical simulation of dissolution of solid particles in fluid flow using the SPH method. Int. J. Numer. Methods Heat Fluid Flow.

[29] Bampton P.A., Dinning P.G., Kennedy M.L., Lubowski D.Z., deCarle D., Cook I.J. (2000). Spatial and temporal organization of pressure patterns throughout the unprepared colon during spontaneous defecation. Am. J. Gastroenterol..

[30] Dinning P.G., Szczesniak M.M., Cook I.J. (2008). Proximal colonic propagating pressure waves sequences and their relationship with movements of content in the proximal human colon. Neurogastroenterol. Motil..

[31] Badley A.D., Camilleri M., Oconnor M.K. (1993). Noninvasive measurement of human ascending colon volume. Nucl. Med. Commun..

[32] Prasanth V.V., Jayaprakas R., Mathew S.T. (2012). Colon specific drug delivery systems: A review on various pharmaceutical approaches. J. Appl. Pharm. Sci..

[33] Lucy L.B. (1977). A numerical approach to the testing of the fission hypothesis. Astronom. J..

[34] Monaghan J.J., Gingold R.A. (1983). Shock simulation by the particle method SPH. J. Comput. Phys..

[35] Monaghan J.J. (2005). Smoothed particle hydrodynamics. Rep. Prog. Phys..

[36] Iacucci M., de Silva S., Ghosh S. (2010). Mesalazine in inflammatory bowel disease: A trendy topic once again?. Can. J. Gastroenterol..

[37] Ye B., van Langenberg D.R. (2015). Mesalazine preparations for the treatment of ulcerative colitis: Are all created equal?. World J. Gastrointest. Pharmacol. Ther..

[38] French D.L., Mauger J.W. (1993). Evaluation of the physicochemical properties and dissolution characteristics of mesalamine—Relevance to controlled intestinal drug-delivery. Pharm. Res..

[39] Hoad C.L., Menys A., Garsed K., Marciani L., Hamy V., Murray K., Costigan C., Atkinson D., Major G., Spiller R.C. (2016). Colon wall motility: Comparison of novel quantitative semi-automatic measurements using cine MRI. Neurogastroenterol. Motil..

[40] Moore J.W., Flanner H.H. (1996). Mathematical comparison of curves with an emphasis on in vitro dissolution profiles. Pharmaceut. Technol..

[41] Shah V.P., Lesko L.J., Fan J., Fleischer N., Handerson J., Malinowski H., Makary M., Duderkirk L., Roy S., Sathe P. (1997). FDA Guidance for industry dissolution testing of immediate release solid oral dosage forms. Dissolut. Technol..

[42] Shah V.P., Tsong Y., Sathe P., Liu J.P. (1998). In vitro dissolution profile comparison—Statistics and analysis of the similarity factor, f2. Pharm. Res..

[43] Birmingham U. University of Birmingham’s BlueBEAR HPC Service. http://www.birmingham.ac.uk.bear.

[44] Ganzenmüller G.C., Steinhauser M.O., Van Liedekerke P. (2011). The Implementation of Smoothed Particle Hydrodynamics in LAMMPS. lammps.sandia.gov/doc/PDF/SPH_LAMMPS_userguide.pdf.

[45] Plimpton S. (1995). Fast parallel algorithms for short-range molecular-dynamics. J. Comput. Phys..

[46] Stukowski A. (2010). Visualization and analysis of atomistic simulation data with OVITO-the Open Visualization Tool. Modell. Simulat. Mater. Sci. Eng..

[47] Hinkle B. (2020). Hatched fill patterns. MATLAB Central File Exchange.

[48] MATLAB (2020). MATLAB 9.9.0.1495850 (R2020b).

[49] Abrahamsson B., Pal A., Sjoberg M., Carlsson M., Laurell E., Brasseur J.G. (2005). A novel in vitro and numerical analysis of shear-induced drug release from extended-release tablets in the fed stomach. Pharm. Res..

[50] Kindgen S., Wachtel H., Abrahamsson B., Langguth P. (2015). Computational fluid dynamics simulation of hydrodynamics and stresses in the PhEur/USP disintegration tester under fed and fasted fluid characteristics. J. Pharm. Sci..

[51] Salehi N., Al-Gousous J., Mudie D.M., Amidon G.L., Ziff R.M., Amidon G.E. (2020). Hierarchical mass transfer analysis of drug particle dissolution, highlighting the hydrodynamics, pH, particle size, and buffer effects for the dissolution of ionizable and nonionizable drugs in a compendial dissolution vessel. Mol. Pharm..

[52] Hopgood M., Reynolds G., Barker R. (2018). Using computational fluid dynamics to compare shear rate and turbulence in the TIM-automated gastric compartment with USP apparatus II. J. Pharm. Sci..

[53] Kukura J., Baxter J.L., Muzzio F.J. (2004). Shear distribution and variability in the USP Apparatus 2 under turbulent conditions. Int. J. Pharm..

[54] Baxter J.L., Kukura J., Muzzio F.J. (2005). Hydrodynamics-induced variability in the USP apparatus II dissolution test. Int. J. Pharm..

[55] Norlander B., Gotthard R., Strom M. (1990). Pharmacokinetics of a 5-aminosalicylic acid enteric-coated tablet in patients with crohns-disease or ulcerative-colitis and in healthy-volunteers. Aliment. Pharmacol. Ther..

[56] Effinger A., O’Driscoll C.M., McAllister M., Fotaki N. (2020). Gastrointestinal diseases and their impact on drug solubility: Crohn’s disease. Eur. J. Pharm. Sci..

[57] Effinger A., O’Driscoll C.M., McAllister M., Fotaki N. (2020). Gastrointestinal diseases and their impact on drug solubility: Ulcerative colitis. Eur. J. Pharm. Sci..

[58] Hebden J.M., Blackshaw P.E., Perkins A.C., Wilson C.G., Spiller R.C. (2000). Limited exposure of the healthy distal colon to orally-dosed formulation is further exaggerated in active left-sided ulcerative colitis. Aliment. Pharmacol. Ther..

[59] Sinha A., Ball D.J., Connor A., Nightingale J., Wilding I. (2003). Intestinal performance of two mesalamine formulations in patients with active ulcerative colitis as assessed by gamma scintigraphy. Pract. Gastroenterol..

[60] Thorpe M.P., Ehrenpreis E.D., Putt K.S., Hannon B. (2009). A dynamic model of colonic concentrations of delayed-release 5-aminosalicylic acid (Asacol). Aliment. Pharmacol. Ther..

